# Methylenetetrahydrofolate reductase gene polymorphisms in the risk of polycystic ovary syndrome and ovarian cancer

**DOI:** 10.1042/BSR20200995

**Published:** 2020-07-17

**Authors:** Ying Xiong, Ce Bian, Xiaojuan Lin, Xiaoli Wang, Kehui Xu, Xia Zhao

**Affiliations:** Department of Gynecology and Obstetrics, Key Laboratory of Obstetrics and Gynecologic and Pediatric Diseases and Birth Defects of Ministry of Education, West China Second University Hospital, Sichuan University, Chengdu 610041, P. R. China

**Keywords:** meta-analysis, methylenetetrahydrofolate reductase, ovarian cancer, polycystic ovary syndrome, polymorphism, variant

## Abstract

Polymorphisms of methylenetetrahydrofolate reductase (MTHFR) in hormone metabolism pathways might cause metabolic disturbances and contribute to the development of polycystic ovary syndrome (PCOS) and ovarian cancer, but the published studies were inconsistent. The aim of the present study was to evaluate the MTHFR C677T (rs1801133) and A1298C (rs1801131) gene polymorphisms in the risk of PCOS and ovarian cancer by meta-analysis. A comprehensive electronic search was conducted in databases for studies published from 1995 to 2020. The pooled ORs were calculated by Revman 5.2 software. Twenty-nine articles including 45 case–control studies were included. We found that MTHFR C677T polymorphisms were correlated with elevated PCOS risk (TT vs. CT+CC: OR = 1.41, 95%CI = 1.20–1.67; TT+CT vs. CC: OR = 1.54, 95%CI = 1.07–2.22; CT vs. CC+TT: OR = 1.18, 95%CI 1.04–1.33; TT vs. CC: OR = 1.47, 95%CI = 1.03–2.11; T vs. C: OR = 1.25, 95%CI = 1.06–1.47), which were more obvious in Middle Eastern subgroup. MTHFR A1298C polymorphisms were also associated with overall PCOS susceptibility (CC vs. AC+AA: OR = 2.55, 95% CI = 1.61–4.03; CC+AC vs. AA: OR = 1.84, 95%CI = 1.04–3.28; CC vs. AA: OR = 2.66, 95%CI = 1.68–4.22; C vs. A: OR = 1.67, 95%CI = 1.03–2.71), which were mainly reflected in Asian subjects. For ovarian cancer, MTHFR C677T polymorphisms were only related with elevated ovarian cancer risk in Asian population, while no significant association was found for A1298C polymorphisms. This meta-analysis suggested that MTHFR C677T and MTHFR A1298C polymorphisms were correlated with elevated PCOS risk. MTHFR C667T only posed a higher risk for ovarian cancer in Asians instead of other populations, while MTHFR A1298C polymorphisms were not related to ovarian cancer risk. Further studies are needed to validate the conclusion.

## Introduction

Polycystic ovary syndrome (PCOS) is one of the most common endocrine malfunctions, reportedly affecting 5–10% of reproductive age women [1]. According to the Rotterdam consensus in 2003, PCOS is featured with oligo- or anovulation, hyperandrogenism and polycystic ovaries [2]. Clinical manifestations of PCOS may include menstrual irregularities, signs of androgen excess, obesity and insulin resistance, with increased risk of Type 2 diabetes and cardiovascular events [3–5]. PCOS impairs female fertility to varying degrees, which remains to be the leading cause for medical assistance. The relationship between PCOS and ovarian cancer has long been controversial. PCOS has been hypothesized to increase ovarian cancer risk through increased androgen exposure in pre-clinical studies [6,7]. Several case–control studies also explored the association of PCOS with ovarian cancer risk, while a recent meta-analysis concluded that there was an increased ovarian cancer risk observed in PCOS population by pooling three individual studies (OR = 1.4; 95% CI = 0.9–2.2) [8]. However, later studies reported no association between self-reported PCOS and ovarian cancer risk. Meanwhile, several studies proposed that an elevation in cancer risk might be relevant to only certain histological subtypes [9,10].

With emerging genetic findings such as homologous repair deficiency in gynecologic malignancies, more and more attention was focused on the genetic root of both diseases. Existing clinical studies suggest that both genetic background and environmental factors with a cluster of metabolic disturbances might contribute to the development of PCOS and ovarian cancer [11,12]. It was also suggested that if the mutation of critical genes in the hormone metabolism pathways could contribute to both diseases individually. It might give us the hint on the pathophysiologic relationship between PCOS and ovarian malignancies. Take homocysteine (Hcy) as an example, it is a sulfur-containing amino acid derived from methionine metabolism and has been proved to be related with insulin resistance and increased risk of cardiovascular diseases in PCOS patients. Elevated plasma level of homocysteine is caused by its deficient transformation, including its transmethylation to methionine, which is regulated by methylenetetrahydrofolate reductase (MTHFR). The MTHFR is an enzyme involved in folate metabolism, and mutations of MTHFR gene would result in reduced activity of the enzyme, thus increasing total Hcy levels in plasma [13,14]. Several gene abnormalities of MTHFR have been explored and two types of SNPs have been found. One is at base position 677 (rs1801133), a C-to-T transition (an alanine to valine substitution). The other is at base position 1298 (rs1801131), an A-to-C transition (a glutamate-to-alanine substitution). It is estimated that approximately 10–15% of Caucasians are homozygous for the TT genotype at positions 677, which is more common in Hispanics (25%) and least common in individuals of African descent (6%) [15]. Another study reported that the overall frequency of the 677TT genotype and 1298CC genotype in the Chinese Han population was 23.2% and 3.9%, respectively [16].

In 1999, Gleuck first reported the association between MTHFR C677T polymorphisms and PCOS, ever since several similar researches have been conducted [17]. In 2014, two independent meta-analyses were published to explore the relationship between MTHFR C677T polymorphisms and PCOS risk but with controversial conclusions [18,19]. One reason of inconsistent conclusions is that neither of the two studies included all current published data. Also, one study categorized subjects from Middle Eastern countries as Caucasian, which might affect the subgroup analysis. Similarly, a certain amount of case–control studies explored the relationship between MTHFR polymorphisms and ovarian cancer. However, the results were conflicting and inconclusive, presumably due to small sample size in each published study while possible selection bias such as ethnicity was unignorable [20,21]. Therefore, in the present study, we conducted a comprehensive meta-analysis by collecting the existing published data to better clarify MTHFR C677T and A1298C polymorphisms in the risk of PCOS and ovarian cancer.

## Materials and methods

### Search for eligible literature

A comprehensive electronic search was performed using PubMed, Embase, Medline (Ovid), Weipu, CNKI and Wanfang databases for studies published from March 1995 to February 2020. The following subject terms and keywords were used: “methylenetetrahydrofolate reductase”, “MTHFR”, “PCOS”, “polycystic ovarian syndrome”, “ovarian cancer” “polymorphism”, “variant” and “mutation”. The search was updated every week until February 20, 2020.

### Inclusion and exclusion criteria

Articles fulfilling the following criteria were included: (i) studied the MTHFR C677T and A1298C polymorphisms in PCOS or ovarian cancer patients, (ii) provided sufficient data in both case and control groups to calculate the odds ratios (ORs) and the corresponding 95% confidence intervals (95% CIs), (iii) mentioned specific diagnostic criteria for PCOS (the NIH criteria or the Rotterdam criteria) and described patients’ symptoms in details, (iv) case–control studies. When duplicate data were present in different articles, only the latest one would be taken into consideration. In addition, Newcastle–Ottawa Scale (NOS) was used to assess the quality of the observational studies included. Three aspects of selection, comparability, and exposure (nine scores in total) were carefully assessed. Studies of moderate or high quality were included (score of 5 or higher). As such, articles that didn’t fulfill the criteria mentioned above were excluded.

### Data extraction

All potential studies were investigated by two independent viewers. The following items were extracted: first author, year of publication, ethnicity, matched parameters, target genotypes, genotyping methods, participant numbers and genotype distributions in cases and controls. Any discrepancies were resolved by discussion with a third reviewer until a consensus was reached.

### Statistical analysis

Meta-analyses were undertaken using the Revman 5.2 softer ware (Cochrane Collaboration, Copenhagen) to calculate the pooled ORs and corresponding 95% CIs. SNPs of MTHFR C677T and A1298C were considered as binary variables. Different contrast models were judged: (i) homozygous mutants contrast, (ii) homozygous and heterozygous mutants contrast, (iii) heterozygous mutants contrast, (iv) homozygous mutants contrast in homozygotes, (v) mutant allelic contrast. Besides the overall comparisons, we also performed subgroup analyses stratified by ethnicities in consideration of population differences. Heterogeneity assumptions were tested using Higgins *I^2^*test. When the *I^2^*value was less than 50%, a fixed-effects model was used otherwise a random-effects model was applied. The *Z* test was performed to determine the significance of the pooled ORs where *P* less than 0.05 was considered statistically significant. The presence of publication bias was evaluated by visually inspecting the asymmetry in funnel plots and Egger’s test. All statistical analyses were performed using Revman 5.2 software (Cochrane Collaboration, Copenhagen, Denmark) except the Egger’s test, which was conducted using STATA 14.0 (StataCorp LP, College Station, TX, U.S.A.).

## Results

### Search results

**Figure 1:**
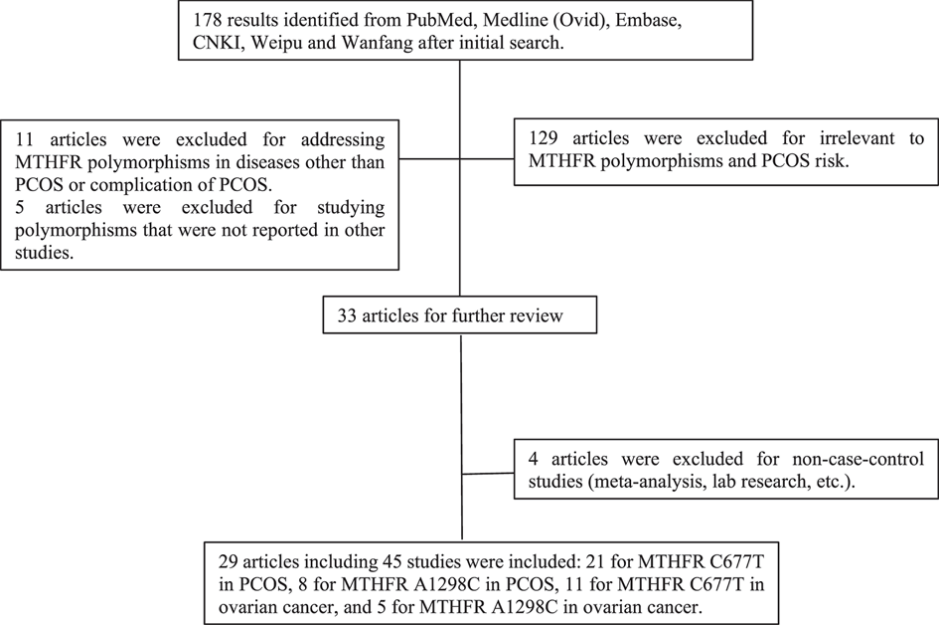
The flow chart of study selection

After primary search, 178 results were retrieved. In our further review, 129 articles were not related to MTHFR polymorphisms and PCOS risk by reading titles and abstracts and thus were excluded. 11 articles focused on MTHFR polymorphisms in different diseases or complication of PCOS like thrombophilia and pregnancy loss. Five studies estimated genetic variants other than C677T and A1298C. Four articles were excluded for non-case–control studies such as meta-analysis and lab research (Figure 1). Among the remaining 29 enrolled articles, 25 were of moderate quality (NOS score of 6 or 7) and 4 were of high quality (NOS score of 8 or 9) therefore were all included in this meta-analysis. By reviewing the genotype counts, 9 articles were found to focus on both C677T and A1298C thus were considered as 18 separate studies. Two articles discussed two different ethnicities thus separate ethnicities were considered as individual studies. One article genotyped subjects from three independent studies and were considered as three studies. Therefore, 45 studies were enrolled for this meta-analysis. We also summarized matched parameters in both case and control groups as shown in Table 1 [17,22–49].

**Table 1:** Characteristics of included studies

First author	Year	Ethnicity	Study disease	MTHFR polymorphism	Genotyping method	Matched parameters	Study quality (NOS)
Carlus	2016	Asian	PCOS	C667T	PCR-RFLP	Age, height, weight, LH, glucose, BMI, LH/FSH	8
Choi	2009	Asian	PCOS	C667T	PCR-RFLP	BMI, weight, waist/hip ratio, FSH, estradiol, prolactin	7
Geng	2016	Asian	PCOS	C667T, A1298C	PCR-RFLP	Age, FSH, prolactin, estradiol	6
Glueck	1999	Caucasian	PCOS	C667T, A1298C	PCR-RFLP	Age, race	7
Idali	2012	Middle Eastern	PCOS	C667T	PCR-RFLP	Unknown	6
Jain	2012	Asian	PCOS	C667T	PCR-RFLP	Age, FSH, TSH, prolactin	7
Jiang	2015	Asian	PCOS	C667T, A1298C	PCR-RFLP	Age	6
Jiao	2018	Asian	PCOS	C667T	PCR-RFLP	Age, estradiol	6
Karadeniz	2010	Middle Eastern	PCOS	C667T	PCR-RFLP	Age, BMI, estradiol, DHEA-S, TSH, prolactin, total cholesterol, triglyceride, LDL-cholesterol	8
Lee	2003	Asian	PCOS	C667T	PCR-RFLP	Unknown	6
Naghavi	2015	Middle Eastern	PCOS	C667T	PCR-RFLP	Age, race	6
Orio	2003	Caucasian	PCOS	C667T	PCR-RFLP	Age, BMI, waist/hip ratio, FSH, prolactin, vitamin B12, folate, homocysteine, fasting glucose	7
Ozegowska	2016	Caucasian	PCOS	C667T	PCR-RFLP	Age, waist/hip ratio, fasting glucose, LDL-C, HDL-C, cholesterol/HDL, SBP, DBP	7
Palep-Singh	2007	Asian& Caucasian	PCOS	C667T, A1298C	PCR-RFLP	South Asian: age, FSH, insulin, cholesterol, LDL. Caucasian: birth weight, waist/hip ratio, right ovarian volume, FSH, testosterone, cholesterol, triglyceride, LDL	
Qi	2015	East Asian	PCOS	C667T, A1298C	PCR-RFLP	Vitamin B12, homocysteine	6
Sills	2001	Caucasian	PCOS	C667T	PCR-RFLP	Age, fasting glucose, androstenedione, DHEA-S, homocysteine	7
Szafarowska	2016	Caucasian	PCOS	C667T, A1298C	PCR-RFLP	Homocysteine, AMH	6
Tsanadis	2002	Caucasian	PCOS	C667T	PCR-RFLP	Age, BMI, DHEA-S, glucose	7
Wu	2016	Asian	PCOS	C667T, A1298C	PCR-RFLP	Age, prolactin, FSH, estradiol, triglyceride	7
Gao	2012	Asian	OC	C667T	PCR-RFLP	Age, BMI, tobacco smoking, alcohol use, menopausal status	7
Jakubowska	2012	Caucasian	OC	C667T	PCR-RFLP	Age, BMI	6
Ozkilic	2016	Middle Eastern	OC	C667T	PCR-RFLP	Age	6
Pawlik	2011	Caucasian	OC	C667T	PCR-RFLP	Age, BMI, FSH, LH, estradiol	6
Prasad	2011	Asian	OC	C667T	MassARRAY	Unknown	6
Song	2012	Asian	OC	A1298C	PCR-RFLP	Age, tobacco use, alcohol use, menopausal status	7
Terry	2010	Caucasian	OC	C667T, A1298C	TaqMan	Age, oral contraceptive use, liveborn number	7
Webb	2011	Caucasian	OC	C667T, A1298C	PCR-PFLP	Age, BMI, oral contraceptive use, energy intake	7
Wu	2007	Asian	OC	C667T	NA	Age, BMI	6
Zhang	2012	Asian	OC	C667T	PCR-RFLP	Tobacco use, alcohol use, menopausal status, hormone replacement therapy	8

Abbreviations: AMH, Anti-Müllerian hormone; BMI, body mass index; DHEA-S, dehydroepiandrosterone sulfate; FSH, follicle-stimulating hormone; HDL-C, high density lipoprotein-cholesterol; LDL-C, low density lipoprotein-cholesterol; LH, luteinizing hormone; MTHFR, methylenetetrahydrofolate reductase; NOS, Newcastle–Ottawa scale; OC, ovarian cancer; PCOS, polycystic ovary syndrome; PCR-RFLP, polymerase chain reaction-restriction fragment length polymorphism; TSH, thyroid-stimulating hormone.

### MTHFR C677T polymorphisms in PCOS

**Figure 2:**
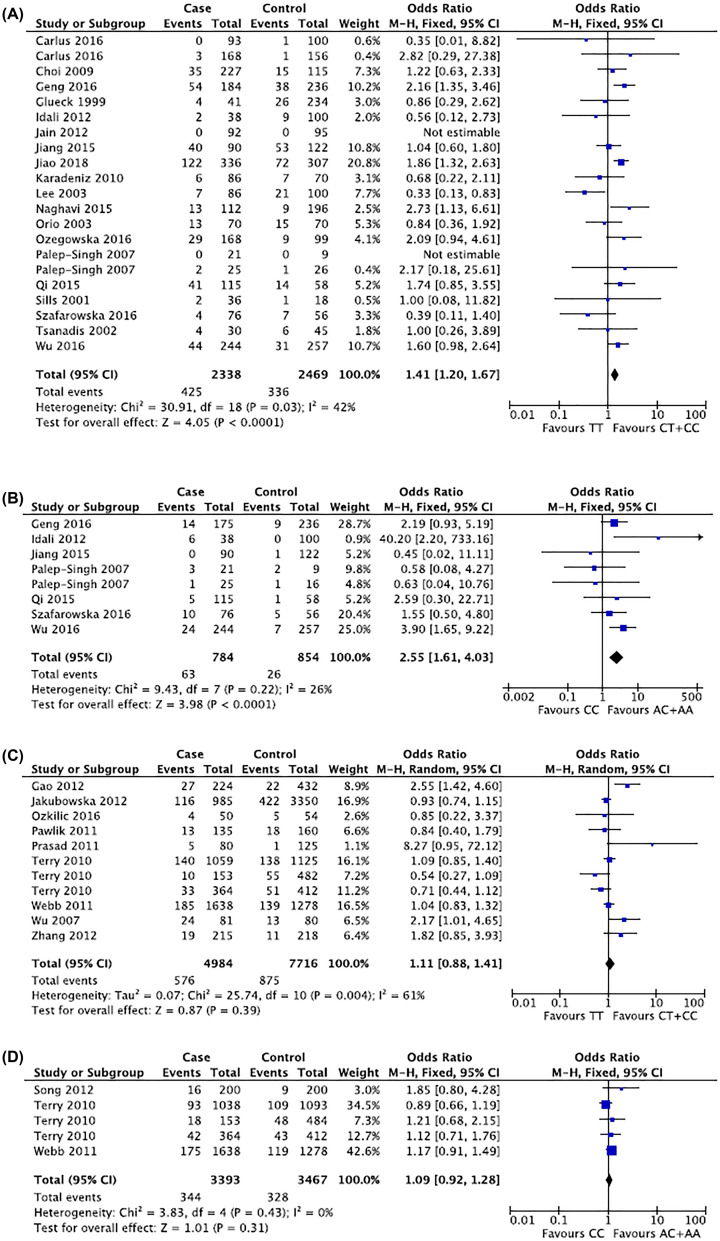
Representative forest plots (**A**) TT vs. CT+CC for MTHFR C667T polymorphisms in PCOS. (**B**) CC vs. AC+AA for A1298C polymorphisms in PCOS. (**C**) TT vs. CT+CC for MTHFR C667T polymorphisms in ovarian cancer. (**D**) CC vs. AC+AA for A1298C polymorphisms in ovarian cancer.

As shown in Tables 2 and 4, the variant allele T has a significant association with the risk of PCOS compared with allele C (T vs. C: OR = 1.25, 95%CI = 1.06–1.47) in random effect model (*I^2^ =* 64%) by pooling 9614 alleles together. In consistent with this, significant association was also found in T containing genotypes (TT+CT or TT) and TT genotypes alone (TT+CT vs. CC: OR = 1.54, 95%CI = 1.07–2.22; TT vs. CC: OR = 1.47, 95%CI = 1.03–2.11; TT vs. CT+CC: OR = 1.41, 95%CI = 1.20–1.67) among 4807 participants (Figure 2A). Interestingly, when comparing heterozygous CT with homozygous TT+CC, the pooled ORs again showed statistical significance (CT vs. CC+TT: OR = 1.18, 95%CI = 1.04–1.33). This indicates that C677T was correlated with elevated risk of PCOS, both in homozygous individuals and heterozygous mutants. Subgroup meta-analyses in stratified ethnicities showed that C677T mutant Middle Eastern population contained higher PCOS risk (TT+CT vs. CC: OR = 2.66, 95%CI = 1.54–4.58; CT vs. CC+TT: OR = 2.64, 95%CI = 1.27–5.49; TT vs. CC: OR = 2.21, 95%CI = 1.16–4.21; T vs. C: OR = 1.82, 95%CI = 1.39–2.37), though only 602 participants were included. No similar tendency was displayed in Asian or Caucasian population.

**Table 2:** Genotype distributions in cases and controls for MTHFR C677T and A1298C polymorphisms in PCOS

Author	Year	Country	Polymorphism	Case			Control		
				AA^∧^	Aa	aa	AA	Aa	aa
Carlus^#^	2016	Asian	C677T	77	16	0	83	16	1
Carlus	2016	Asian	C677T	132	33	3	126	29	1
Choi	2009	Asian	C677T	67	125	35	33	67	15
Geng	2016	Asian	C677T	51	79	54	102	96	38
Glueck	1999	Caucasian	C677T	14	23	4	119	89	26
Idali	2012	Middle Eastern	C677T	17	19	2	66	25	9
Jain	2012	Asian	C677T	76	16	0	82	13	0
Jiang	2015	Asian	C677T	13	37	40	13	56	53
Jiao	2018	Asian	C677T	52	162	122	96	139	72
Karadeniz	2010	Eastern	C677T	15	65	6	35	28	7
Lee	2003	Asian	C677T	33	46	7	27	52	21
Naghavi	2015	Middle Eastern	C677T	61	38	13	136	51	9
Orio	2003	Caucasian	C677T	16	41	13	17	38	15
Ozegowska	2016	Caucasian	C677T	87	52	29	53	37	9
Palep-Singh*	2007	Asian	C677T	14	7	0	9	0	0
Palep-Singh	2007	Caucasian	C677T	11	12	2	10	15	1
Qi	2015	Asian	C677T	14	60	41	21	23	14
Sills	2001	Caucasian	C677T	25	9	2	8	9	1
Szafarowska	2016	Caucasian	C677T	33	39	4	19	30	7
Tsanadis	2002	Caucasian	C677T	12	14	4	20	19	6
Wu	2016	Asian	C677T	94	106	44	122	104	31
Geng	2016	Asian	A1298C	104	57	14	157	70	9
Idali	2012	Eastern	A1298C	12	20	6	94	6	0
Jiang	2015	Asian	A1298C	66	24	0	98	23	1
Palep-Singh	2007	Asian	A1298C	9	9	3	0	7	2
Palep-Singh*	2007	Caucasian	A1298C	14	10	1	10	5	1
Qi	2015	Asian	A1298C	71	39	5	43	14	1
Szafarowaska	2016	Caucasian	A1298C	40	26	10	34	17	5
Wu	2016	Asian	A1298C	143	77	24	166	84	7

∧ ‘A’ represents wild-type allele and ‘a’ represents mutant allele.

# Study separately enrolled two sub-populations of Indian and were thus considered as two studies.

* Study separately enrolled Asian and Caucasian and were thus considered as two studies.

### MTHFR A1298C polymorphisms in PCOS

The result of the association between MTHFR A1298C and PCOS risk was as followed. About 7 articles including 8 studies with 784 PCOS patients and 854 healthy controls were pooled. Except the heterozygous mutant comparison that presented an insignificant OR, others showed significant outcome between A1298C mutation and elevated PCOS risk (CC vs. AC+AA: OR = 2.55, 95% = 1.61–4.03; CC+AC vs. AA: OR = 1.84, 95%CI = 1.04–3.28; CC vs. AA: OR = 2.66, 95%CI = 1.68–4.22; C vs. A: OR = 1.67, 95%CI = 1.03–2.71) (Figure 2B). When stratified by ethnicity, only Asians shared consistent results with the overall population (CC vs. AC+AA: OR = 2.46, 95% = 1.44–4.22; CC+AC vs. AA: OR = 1.33, 95%CI = 1.05–1.67; CC vs. AA: OR = 2.43, 95%CI = 1.41–4.18; C vs. A: OR = 1.39, 95%CI = 1.14–1.69). Notably, fixed-effect model was conducted in both Asian and Caucasian comparisons but random-effect model was used for overall population. This might suggest the consistency among the same ethnicity but differences among different populations might exist. The above data indicated that MTHFR A1298C posed a higher risk for PCOS in overall population, particularly in Asians instead of other populations. The detailed results were summarized in Tables 2 and 5.

### MTHFR C677T and A1298C polymorphisms in ovarian cancer

Genetic distributions and pooled ORs were shown in Tables 3 and 6. Eleven studies including 12,700 participants were evaluated for C677T polymorphisms in ovarian cancer risk. While the overall group analysis presented no relationship between C-to-T mutation and ovarian cancer susceptibility (Figure 2C), the stratified group analysis of 1455 Asian subjects indicated an increased cancer risk (TT vs. CT+CC: OR = 2.35, 95%CI = 1.59–3.48; TT+CT vs. CC: OR = 1.49, 95%CI = 1.19–1.86; TT vs. CC: OR = 2.84, 95%CI = 1.88–4.31; T vs. C: OR = 1.49, 95%CI = 1.26–1.77). Since the Asian population only consisted of a minority among the overall group subjects, it should be noted that the overall group conclusion might be changed when sample sizes of different ethnicities vary. In general, MRHFR C677R polymorphisms was associated with increased cancer risk in Asian population.

**Table 3:** Genotype distributions in cases and controls for MTHFR C677T and A1298C polymorphisms in ovarian cancer

Author	Year	Country	Polymorphism	Case			Control		
				AA^∧^	Aa	aa	AA	Aa	aa
Gao	2012	Asian	C677T	97	100	27	232	178	22
Jakubowska	2012	Caucasian	C677T	423	446	116	1447	1481	422
Ozkilic	2016	Middle Eastern	C677T	18	28	4	19	30	5
Pawlik	2011	Caucasian	C677T	67	55	13	63	79	18
Prasad	2011	Asian	C677T	72	3	5	116	8	1
Terry^#^	2010	Caucasian	C677T	427	492	140	499	488	138
Terry	2010	Caucasian	C677T	71	72	10	210	217	55
Terry	2010	Caucasian	C677T	164	167	33	193	168	51
Webb	2011	Caucasian	C677T	744	709	185	571	568	139
Wu	2007	Asian	C677T	17	40	24	32	35	13
Zhang	2012	Asian	C677T	102	94	19	115	92	11
Song	2012	Asian	A1298C	107	77	16	112	79	9
Terry	2010	Caucasian	A1298C	515	430	93	534	450	109
Terry	2010	Caucasian	A1298C	68	67	18	236	200	48
Terry	2010	Caucasian	A1298C	173	149	42	189	180	43
Webb	2011	Caucasian	A1298C	770	693	175	598	561	119

∧ ‘A’ represents wild-type allele and ‘a’ represents mutant allele.

# Study separately genotyped subjects from three studies, the New England Case Control Study (NEC), Nurses’ Health Study (NHS), and Mayo Clinic Ovarian Cancer Case Control Study (MAY), thus were considered as three studies.

**Table 4:** Summary of different comparative results for MTHFR C677T polymorphisms in PCOS

Genotypes	Overall and subgroup	Participants	OR and 95%CI	*Z* value	*P* value	*I^2^* (%)	Effect model^#^
TT vs. CT+ CC	Overall	4,807	1.41 [1.20, 1.67]	4.05	0.01	42	F
	Asian	3,211	1.38 [0.99, 1.92]	1.92	0.05	55	R
	Caucasian	994	1.09 [0.72, 1.65]	0.39	0.7	0	F
	Middle Eastern	602	1.13 [0.39, 3.32]	0.23	0.82	60	R
TT+ CT vs. CC	Overall	4,807	1.54 [1.07, 2.22]	2.31	0.02	85	R
	Asian	3,211	1.76 [0.99, 3.13]	1.62	0.05	90	R
	Caucasian	994	1.03 [0.78, 1.36]	0.21	0.84	29	F
	Middle Eastern	602	2.66 [1.54, 4.58]	3.52	0.01	53	R
CT vs. CC+ TT	Overall	4,807	1.18 [1.04, 1.33]	2.64	0.01	49	F
	Asian	3,211	1.10 [0.95, 1.27]	1.25	0.21	0	F
	Caucasian	994	0.99 [0.75, 1.31]	0.05	0.96	40	F
	Middle Eastern	602	2.64 [1.27, 5.49]	2.6	0.01	74	R
TT vs. CC	Overall	2,872	1.47 [1.03, 2.11]	2.1	0.04	59	R
	Asian	1,929	1.58 [0.92, 2.72]	1.67	0.09	75	R
	Caucasian	567	1.14 [0.72, 1.81]	0.58	0.56	0	F
	Middle Eastern	376	2.21 [1.16, 4.21]	2.41	0.02	0	F
T vs. C	Overall	9,614	1.25 [1.06, 1.47]	0.64	0.01	64	R
	Asian	6,422	1.27 [1.01, 1.61]	2.03	0.04	74	R
	Caucasian	1,988	1.04 [0.85, 1.27]	0.34	0.73	19	F
	Middle Eastern	1,204	1.82 [1.39, 2.37]	4.38	0.01	0	F

# Effect model includes fixed-effect model (F) and random-effect model (R).

**Table 5:** Summary of different comparative results for MTHFR A1298C polymorphisms in PCOS

Genotypes	Overall and subgroup	Participants	OR and 95%CI	*Z* value	*P* value	*I^2^* (%)	Effect model^#^
CC vs. AC+AA	Overall	1,638	2.55 [1.61, 4.03]	3.98	0.01	26	F
	Asian	1,327	2.46 [1.44, 4.22]	3.28	0.01	6	F
	Caucasian	173	1.37 [0.48, 3.90]	0.59	0.55	0	F
CC+AC vs. AA	Overall	1,638	1.84 [1.04, 3.28]	2.09	0.04	81	R
	Asian	1,327	1.33 [1.05, 1.67]	2.4	0.02	14	F
	Caucasian	173	1.37 [0.74, 2.54]	1.01	0.31	0	F
AC vs. AA+CC	Overall	1,638	1.52 [0.89, 2.57]	1.54	0.12	78	R
	Asian	1,327	1.11 [0.88, 1.41]	0.87	0.39	34	F
	Caucasian	173	1.25 [0.66, 2.39]	0.69	0.49	0	F
CC vs. AA	Overall	1,150	2.66 [1.68, 4.22]	4.15	0.01	49	F
	Asian	923	2.43 [1.41, 4.18]	3.2	0.01	39	F
	Caucasian	115	1.51 [0.52, 4.42]	0.75	0.45	0	F
C vs. A	Overall	3,276	1.67 [1.03, 2.71]	2.07	0.04	83	R
	Asian	2,654	1.39 [1.14, 1.69]	3.3	0.01	34	F
	Caucasian	346	1.31 [0.80, 2.14]	1.08	0.28	0	F

# Effect model includes fixed-effect model (F) and random-effect model (R).

**Table 6:** Summary of different comparative results for MTHFR C677T polymorphisms in ovarian cancer

Genotypes	Overall and subgroup	Participants	OR and 95%CI	*Z* value	*P* value	*I^2^* (%)	Effect model^#^
TT vs. CT+ CC	Overall	12,700	1.11 [0.88, 1.41]	0.87	0.39	61	R
	Asian	1,455	2.35 [1.59, 3.48]	4.3	0.01	0	F
	Caucasian	11,141	1.82 [0.85, 3.93]	0.72	0.47	16	F
TT+ CT vs. CC	Overall	12,700	1.06 [0.99, 1.15]	1.58	0.11	49	F
	Asian	1,455	1.49 [1.19, 1.86]	3.44	0.01	3	F
	Caucasian	11,141	1.02 [0.94, 1.10]	0.46	0.64	32	F
CT vs. CC+ TT	Overall	12,700	1.05 [0.97, 1.13]	1.17	0.24	0	F
	Asian	1,455	1.11 [0.88, 1.39]	0.89	0.37	0	F
	Caucasian	11,141	1.04 [0.96, 1.13]	0.92	0.36	22	F
TT vs. CC	Overall	7,150	1.18 [0.89, 1.55]	1.16	0.24	68	R
	Asian	915	2.84 [1.88, 4.31]	4.93	0.01	0	F
	Caucasian	6,199	0.97 [0.85, 1.11]	0.41	0.68	25	F
T vs. C	Overall	25,400	1.08 [0.96, 1.21]	1.31	0.19	66	R
	Asian	2,910	1.49 [1.26, 1.77]	4.6	0.01	10	F
	Caucasian	22,282	1.00 [0.94, 1.06]	0	1	29	F

# Effect model includes fixed-effect model (F) and random-effect model (R).

The results of MTHFR A1298C polymorphisms in ovarian cancer were straightforward (Figure 2D). The meta-analysis failed to show significant association between variant genotypes (or alleles) and ovarian cancer susceptibility in corresponding effect models, neither in overall group analysis nor Caucasian subgroup analysis (Tables 3 and 7).

**Table 7:** Summary of different comparative results for MTHFR A1298C polymorphisms in ovarian cancer

Genotypes	Overall and subgroup	Participants	OR and 95%CI	*Z* value	*P* value	*I^2^* (%)	Effect model^#^
CC vs. AC+AA	Overall	6,860	1.09 [0.92, 1.28]	1.01	0.31	0	F
	Caucasian	6,460	1.06 [0.90, 1.26]	0.74	0.46	0	F
CC+AC vs. AA	Overall	6,860	1.00 [0.91, 1.10]	0.06	0.95	0	F
	Caucasian	6,460	0.99 [0.90, 1.09]	0.19	0.85	0	F
AC vs. AA+CC	Overall	6,860	0.97 [0.88, 1.07]	0.67	0.5	0	F
	Caucasian	6,460	0.97 [0.88, 1.07]	0.64	0.52	0	F
CC vs. AA	Overall	3,974	1.08 [0.91, 1.27]	0.85	0.4	0	F
	Caucasian	3,730	1.05 [0.88, 1.25]	0.57	0.57	0	F
C vs. A	Overall	13,720	1.02 [0.94, 1.09]	0.41	0.68	0	F
	Caucasian	12,920	1.01 [0.93, 1.09]	0.19	0.85	0	F

# Effect model includes fixed-effect model (F) and random-effect model (R).

### Publication bias

**Figure 3:**
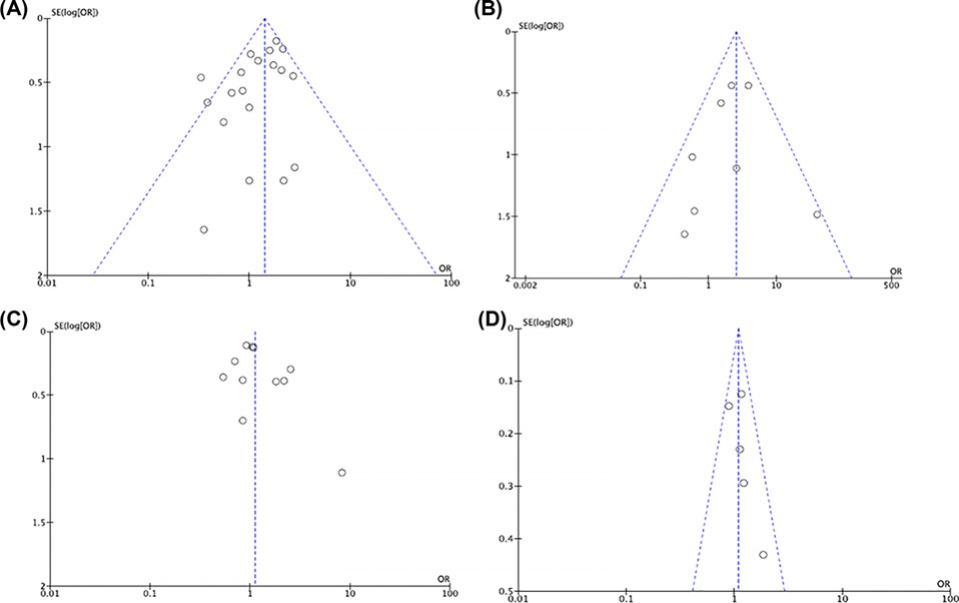
Representative funnel plots (**A**) TT vs. CT+CC for MTHFR C667T polymorphisms in PCOS. (**B**) CC vs. AC+AA for A1298C polymorphisms in PCOS. (**C**) TT vs. CT+CC for MTHFR C667T polymorphisms in ovarian cancer. (**D**) CC vs. AC+AA for A1298C polymorphisms in ovarian cancer.

The shapes of the funnel plots appeared to be symmetrical in all genetic comparisons, indicating the lack of publication bias and the reliability of the meta-analysis in both overall and subgroups (Figure 3). We further performed Egger’s tests in the analyses that proposed significant ORs. The results demonstrated no significant publication bias (*P*>0.05, data not shown).

## Discussion

Polycystic ovary syndrome consists a heterogeneous endocrinological disorder, which is characterized by oligo- or anovulation, hyperandrogenism and polycystic ovaries [50]. These pathophysiological malfunctions are associated with clinical manifestations like oligomenorrhea, amenorrhea, infertility, hirsutism, obesity, acne, Type 2 diabetes mellitus, skin hyperpigmentation, etc [51,52]. The long term of treatments and the consequential infertility seriously affect women’s living qualities, even though many symptoms could be meliorated by oral contraceptive and metformin. Contradictory evidence exists regarding PCOS and risk of ovarian cancer. Some studies suggested that self-reported PCOS was related to higher risk of ovarian cancer when age was adjusted, while others concluded no significant association between the two diseases. Moreover, several studies reported that PCOS might decrease the risk of ovarian cancer because the use of oral contraceptives were common among such patients [53,54]. The etiology of PCOS and ovarian cancer involve multiple genetic and epigenetic alterations that cause changes in metabolic enzymes such as MTHFR. It has been reported that polymorphisms of the MTHFR gene might reduce MTHFR enzyme activity. As a result, the concentration of Hcy in plasma would be increased, which is commonly discovered in PCOS and ovarian cancer patients. Even recently, several trials have explored the efficacy of folate receptor antagonist in ovarian cancer [55]. This clinically reflects the significance of folate metabolic pathways in ovarian carcinogenesis. Therefore, it is assumed that the abnormality (including several polymorphisms) of MTHFR, which is a key component of the folate pathways, could contribute to increased PCOS and ovarian cancer risk.

In the present study, we performed an updated meta-analysis to explore MTHFR C667T and A1298C polymorphisms in the PCOS and ovarian cancer risk. Twenty-nine articles including 45 case–control studies were included. We found that MTHFR C677T polymorphisms were correlated with elevated PCOS risk, which were more obvious in Middle Eastern subgroups. Also, MTHFR A1298C polymorphisms were associated with overall PCOS risk, which were mainly reflected in Asians. For ovarian cancer, MTHFR C677T polymorphisms were only related with elevated ovarian cancer risk in Asians, while no significant association was found for A1298C polymorphisms. Several points could be noted based on current results. First, both MTHFR C677T and MTHFR A1298C polymorphisms were correlated with elevated PCOS risk. Although polymorphisms of A1298C seem to be less prevalent than C677T, the current findings suggest that any impairment on folate methylation could impact the development of PCOS, regardless of its frequency [16]. As such, new genetic abnormalities in folate metabolism pathways are of great potential in unrevealing the critical pathogenic factor of PCOS. Second, ethnicity difference seems to have an impact on the disease susceptibility. Despite confounding factors that could not be excluded from the study, such findings may indicate that different genetic components could play different extends of roles in the disease development, leading to different manifestations. For instance, it was reported that East Asian patients with PCOS were more likely to have diabetes compared with Caucasian patients [56]. Thus, continuous genome-based studies are needed to personalize the diagnosis and management of PCOS across different ethnicities. Third, the relationship between MTHFR and ovarian cancer is less conclusive compared with PCOS. While both polymorphisms are associated with increased PCOS risk in overall population, significant result was only observed in MTHFR C667T Asian subgroup for ovarian cancer. PCOS has been hypothesized to increase ovarian cancer risk through androgen exposure in pre-clinical settings and oral contraceptive use has been proved to reduce the risk of ovarian cancer, suggesting the potential metabolic relationship between PCOS and ovarian cancer [57,58]. However, it is hard to conclude a concrete relationship between PCOS and ovarian cancer under the mechanism of MTHFR gene polymorphisms based on the current results. Whereas such findings could not rule out the potential relationship between PCOS and ovarian cancer risk. In a population-based, case–control study of 476 subjects with histologically confirmed epithelial ovarian cancer, Schildkraut et al. found that ovarian cancer risk was found to increase 2.5-fold (95% CI: 1.1–5.9) among women with PCOS [59]. Moreover, the possibility that the pathogenesis of PCOS and ovarian cancer might be interacted on the pathways of hormone metabolism still exist. In fact, recent identification of several proteins overexpressed in both PCOS and ovarian cancer, including calreticulin, fibrinogen, superoxide dismutase, and vimentin gave us a clue on the two diseases and even promising subgroup identification of ovarian cancer based on PCOS development. As such, whether MTHFR polymorphisms played a role in this intriguing contribution remains to be further studied.

Despite our efforts to pool the results of currently published studies, some disadvantages of the present meta-analysis still existed. First, the number of enrolled studies was so far the largest among all relevant meta-analysis but was still limited. It is possible that the results of further investigations and unpublished articles might be different from the present conclusion, thus cautions should be paid to explain the results. Second, this meta-analysis was based on unadjusted estimations. Although matched parameters were carefully reviewed, it is known that some unreported risk factors like family history and genetic information (e.g. BRCA mutation, HRD status) were also important in the development of PCOS or ovarian cancer [60]. Meanwhile, no histological subtypes of ovarian cancer were provided in the source studies. Since it is reported that an elevation in ovarian cancer risk might be relevant to only certain histological subtypes [9,10], the absence or enrichment of certain types of ovarian cancer in the present study might not reveal the real-world disease landscape. All those confounding factors mentioned above might affect the validity of the results. In addition, MTHFR polymorphisms and elevated homocysteine levels may increase risks of several other diseases such as thromboembolism, endometrial cancer, hypertension, diabetes, etc. Such conditions themselves may interact with diet, concurrent medication and lifestyle, thus affecting the susceptibility of PCOS and ovarian cancer. Third, we confirmed the risk prone effects of MTHFR polymorphisms in Middle Eastern population for PCOS and particular MTHFR A1298C polymorphisms in Asian population for ovarian cancer. The ethnicities should be deliberately illustrated because of two reasons. One is that the ethnicity distribution in the overall group did not reflect the real-world percentages. The sample size changes due to future publications in Asian population for C667T in ovarian cancer risk might affect the final readout of overall group analysis. The other is that only Turkish and Iranian were included in the Middle Eastern subgroup and most of the stratified Asians were Chinese, while other populations such as Hispanics and Black were not within this discussion. Thus, studies enrolling diverse ethnicities were required.

## Conclusion

To our knowledge, the present study was the most updated meta-analysis exploring the association between MTHFR C677T and A1298C polymorphisms and the PCOS and ovarian cancer. It was also the first study to explore the MTHFR polymorphisms in both diseases. Although it was hard to conclude a concrete association between PCOS and ovarian cancer under the mechanism of MTHFR gene polymorphisms, the present study suggested that MTHFR C677T and A1298C polymorphisms were correlated with elevated PCOS risk while MTHFR C677T polymorphisms only posed a higher risk for ovarian cancer in Asians. Further studies are needed to validate the conclusion.

## Abbreviations

AMH: Anti-Müllerian hormone
BMI: body mass index
DHEA-S: dehydroepiandrosterone sulfate
FSH: follicle-stimulating hormone
HDL-C: high density lipoprotein-cholesterol
LDL-C: low density lipoprotein-cholesterol
LH: luteinizing hormone
MTHFR: methylenetetrahydrofolate reductase
NOS: Newcastle–Ottawa scale
